# Developing Family Healthware, a Family History Screening Tool to Prevent Common Chronic Diseases

**Published:** 2008-12-15

**Authors:** Paula W. Yoon, Maren T. Scheuner, Cynthia Jorgensen, Muin J. Khoury

**Affiliations:** Centers for Disease Control and Prevention; Rand Corporation, Santa Monica, California; Centers for Disease Control and Prevention, Atlanta, Georgia; Centers for Disease Control and Prevention, Atlanta, Georgia

## Abstract

Family health history reflects the effects of genetic, environmental, and behavioral factors and is an important risk factor for a variety of disorders including coronary heart disease, cancer, and diabetes. In 2004, the Centers for Disease Control and Prevention developed Family Healthware, a new interactive, Web-based tool that assesses familial risk for 6 diseases (coronary heart disease, stroke, diabetes, and colorectal, breast, and ovarian cancer) and provides a "prevention plan" with personalized recommendations for lifestyle changes and screening. The tool collects data on health behaviors, screening tests, and disease history of a person's first- and second-degree relatives. Algorithms in the software analyze the family history data and assess familial risk based on the number of relatives affected, their age at disease onset, their sex, how closely related the relatives are to each other and to the user, and the combinations of diseases in the family. A second set of algorithms uses the data on familial risk level, health behaviors, and screening to generate personalized prevention messages. Qualitative and quantitative formative research on lay understanding of family history and genetics helped shape the tool's content, labels, and messages. Lab-based usability testing helped refine messages and tool navigation. The tool is being evaluated by 3 academic centers by using a network of primary care practices to determine whether personalized prevention messages tailored to familial risk will motivate people at risk to change their lifestyles or screening behaviors.

## Introduction

In 2002, the Centers for Disease Control and Prevention (CDC) launched a public health initiative to evaluate the use of family history information for assessing risk for common diseases and influencing early detection and prevention strategies (1). A multidisciplinary work group reviewed the evidence for family history as a risk factor for common diseases (2-9), assessed existing tools for gathering and interpreting family history data, and recommended the development of a new family history tool. We describe the development of Family Healthware, including the need for the tool, underlying design principles, tool components, familial risk stratification, formative research, and the evaluation plan for determining the tool's validity and utility.

### Family history and health

Family history is a significant and prevalent risk factor for many common diseases, and for most diseases, it is reported with a high degree of accuracy. (The Table reviews associated risk, prevalence, and accuracy of self-reports of family history). For the common chronic diseases that are typically multifactorial in nature (and only rarely genetically inherited), family history of disease reflects the effects of genetic and nongenetic risk factors (eg, exposures, behaviors, cultural factors) shared by affected family members.

Knowledge of the family history can guide risk-specific recommendations for disease management and prevention, including referral to a specialist for evaluation and possible testing. For people suspected of having rare Mendelian disorders, recognizing personal and family history characteristics is crucial for determining which patients should be offered genetic testing. Prevention strategies for people with increased familial risk of common diseases could include lifestyle changes; screening at earlier ages, more frequently, and using more intensive methods than those used for average-risk individuals; use of chemoprevention; and for those at highest risk, prophylactic procedures and surgeries. Data about effectiveness of these strategies for high-risk individuals are accumulating (31,32).

### Tools for gathering and interpreting family history

Americans are not in the habit of collecting and documenting their family health history, although a survey found that 96% considered knowledge of family history important to their personal health (33). Physicians also perform poorly with respect to collecting family health history from their patients and in interpreting and using family history to recommend risk-specific interventions (34-37). Clinicians may neglect family history because of the amount of time required to collect the information and possible lack of compensation for these efforts, as well as concerns about their ability to interpret such information and accurately counsel patients about their risk (38,39).

These barriers could be largely overcome through use of electronic family history tools that can 1) collect relevant personal and family health history in a structured, codified format; 2) organize the data into a usable form such as a graphic display; 3) interpret the familial risk and recognize patterns of disease suggestive of inherited susceptibilities; 4) provide input to overall risk assessment; and 5) recommend interventions tailored to the familial risk and personal factors. The tools and methods available for gathering and interpreting family histories are lacking many of these capabilities. Of the available family history tools designed to interpret familial risk, most have been developed for cancer, particularly breast and colorectal cancer (40,41). Generally, these familial risk assessment tools are clinical scoring methods, or they use clinical criteria to define the familial risk level. A limitation of these methods is that it may be impossible to classify individuals whose family histories do not fit the criteria, which is problematic for a tool designed for the public or one that provides clinical decision support.

## Methods

Family Healthware was developed to address the needs of health professionals who value accurate family history information for risk assessment but have limited time and resources for collecting and interpreting that information, and the public who believe their family history is important but for the most part do not actively gather and record that information. The design and content of Family Healthware were specified by the multidisciplinary work group, and the software was developed by a major commercial communications firm with support from a software development company. The tool automates the process of assessing family history risk and personalizing disease prevention strategies, and it provides educational resources for health professionals and consumers.

**Design principles**

The work group reviewed family history tools that were being used or developed, and identified tool characteristics that enable use for risk assessment and disease prevention, as well as those that make the tools cumbersome or impractical for use by the public and health professionals. From this assessment, we specified several key design principles for a new family history tool that focused on health promotion and prevention of common chronic diseases:

- Self-administered
- Flexible and adaptable to different settings (eg, stand-alone tools for the consumer, integrated within electronic medical record or public health surveillance systems)
- Simple, easy to use, and designed to collect relevant data for familial risk assessment
- Interpretation of familial risk is based on algorithms
- Evidence-based prevention strategies recommended are appropriate for the familial risk

A critical first step in developing Family Healthware was to decide which diseases to include. Criteria that could be used to guide the selection of diseases (1) were adapted and augmented from screening criteria first established by the World Health Organization (42) and later updated by, among others, the Council of Europe (43):

- The disease poses a substantial public health burden.
- A well-defined case definition is available.
- Relatives have a high awareness of disease status.
- The disease is accurately reported by relatives.
- Family history is an established risk factor.
- Effective interventions exist for primary and secondary disease prevention and are specific to the familial risk.

A list of diseases was compiled from tools being used in primary care. We applied the inclusion criteria to a list of nearly 50 diseases; 15 met many of the criteria (coronary heart disease [CHD], sudden unexpected death, stroke, hypertension, diabetes, blood clots in lungs or legs, emphysema/lung disease, kidney disease, breast cancer, ovarian cancer, prostate cancer, colorectal cancer, endometrial cancer, thyroid cancer, and kidney cancer). However, very few met all of the criteria. For example, cancers of the female pelvic organs such as endometrial and ovarian tend to be inaccurately reported by relatives (9); however, ascertaining a family history of these cancers is important for recognizing hereditary nonpolyposis colorectal cancer and hereditary breast and ovarian cancer, respectively. The last criterion, "effective interventions exist for primary and secondary disease prevention and are specific to the familial risk," presented the greatest challenge for most diseases. However, as data accumulate on the validity and utility of using family history as a screening tool, the availability of risk-specific interventions may change. The current version of Family Healthware includes 6 diseases — CHD, stroke, diabetes, and breast, ovarian, and colorectal cancer. The work group decided that the number of diseases should be limited in the first version of the tool to facilitate its testing and evaluation in different population-based settings.

**Algorithms that interpret familial risk**

The familial risk stratification (FRS) method integrated within Family Healthware is based on a framework that represents the elements of a pedigree including at a minimum the index case and the first- and second-degree relatives. In this framework, absence (*no* or *don't know* responses) or presence of a disease and age of disease onset are considered for every combination of personal and family medical history and are assigned a weak, moderate, or strong value according to rules derived from empirical data. When such data are unavailable, general principles of familial risk assessment are followed (44). Rules regarding lineage (ie, maternal, paternal, or nuclear) of affected family members are also applied, thus allowing for recognition of hereditary syndromes that follow Mendelian modes of inheritance (eg, autosomal dominant, autosomal recessive, X-linked, mitochondrial). Usually, a weak familial risk is assigned if there is no family history or if there is late-onset disease in only 1 second-degree or more distant relative from 1 or different sides of the family. Moderate familial risk is generally assigned if there is only 1 first-degree relative with late-onset disease or 2 second-degree relatives from the same lineage with late-onset disease. Strong familial risk is generally assigned if there is a first-degree relative with early-onset disease, when multiple relatives are affected, or when a hereditary syndrome is suspected. For most common chronic diseases, a moderate familial risk is associated with about a 2-fold increase in risk over a weak familial risk, and a strong familial risk is associated with about a 3-fold or greater increase (44).

**Evidence-based prevention messages**

In the early phases of design, the work group specified that Family Healthware should collect only family history information. A report generated by the tool would describe the level of risk for each disease and include general messages about health behaviors and screening tests to prevent the disease or detect it early. A review of the literature suggested, however, that prevention recommendations tailored to patients' level of disease risk was associated with increased screening for higher-risk patients (45,46), though evidence to suggest that tailored messages influenced health behaviors was limited (47). The work group decided that the tool would be more valuable if the health behavior and screening messages could be tailored not only to family history but also to the user's lifestyle and health habits. Therefore, in addition to collecting family history information, the tool was designed to collect information about health behaviors and screening tests relevant to the 6 diseases. A second set of algorithms was added to assign prevention messages based on the familial risk level, answers to questions about screening tests and health behaviors, and sex and age.

**Development of the tool interface**

We developed the interface for the tool through an iterative process by using a combination of formative research techniques. First, we reviewed the concept to make sure it was consistent with scientific evidence and clinical practice. Existing family history tools, both paper-based and electronic, were assessed to identify best practices and data collection gaps. Next, we tested several design aspects of the tool with a series of consumer focus groups. For example, is it easier for a person to focus on a specific disease and recall affected relatives or to focus on specific relatives and recall the diseases they have had? We found that, generally speaking, focus group participants were familiar with family history and could easily recall completing a written family history during a primary care visit. Few people, however, could recall updating their family history on subsequent visits. Knowledge of relatives' health history varied greatly; common explanations include premature death, distant or estranged relationships, large age differences, confusion over cause of death, and little communication over personal health matters.

Most participants supported the idea of a computerized family history tool, even though their personal comfort and experience with computers varied. They preferred a tool provided by their personal physician or health care organization, rather than outside groups or insurance providers. Perhaps the most significant finding was that the focus group participants, like the work group, stressed the importance of lifestyle in disease prevention and strongly advocated for lifestyle recommendations to be added to the tool. We tested potential labels for 3 levels of familial risk by using wire frames, or paper drawings of screen layouts, with 8 focus groups.

The participants found the information that was presented relevant, but they wanted even more specific and detailed screening and lifestyle recommendations. For example, they wanted to know which screening tests were best for them, why, and how often they should get them. Reactions to the lifestyle messages were similar. Most reported familiarity with the messages (eg, eat at least 5 servings of fruits and vegetables daily, lose weight, quit smoking) but again requested more specific and directive information (eg, which fruits and vegetables to eat, how much weight to lose).

Reactions to the labels that described familial risk were more complicated. First, participants were confused about familial risk versus *overall* risk for a disease. They preferred to know their overall risk and wanted the tool to provide that assessment. Many people interpreted "no familial risk" to mean "no disease risk." In addition, there was some confusion about terms such as *average* and *moderate*, and using a qualifying term (eg, *very*) with a label such as *high* caused great concern. As a result of our testing, we decided to use "strong," "moderate," and "weak" paired with "family history" to describe familial risk — for example, a "weak family history" for diabetes. We believe these labels are valid, represent a familiar rating system, accurately describe familial risk, and limit confusion about the concept of familial risk versus overall risk for a disease.

**Quality assurance testing**

To assess the consistency and logic of the programming for the FRS method, we used a process called "harness testing," whereby the FRS framework and accompanying rules for interpreting the familial risk were tested by computer-generated family history scenarios of every possible combination of events. This process was followed by an internal expert review that checked for consistency between the output of the tool and the written programmed directions and by heuristic testing with internal and external experts. Experts in computer application design and navigation used the tool and advised how best to design the various navigational and visual elements of the tool.

**Usability testing**

An independent research firm conducted usability testing. The Figure is an example of a screen shot from the tool, with notations made after the testing process. For each of 2 rounds, 8 men and women older than 45 with some family history of 1 of the 6 diseases assessed by the tool participated in a 1-on-1 usability test conducted in a computer laboratory. An experienced interviewer guided each person through the application, soliciting feedback on key aspects. Questions were designed to generate users' reactions, assessments, difficulties in tool use, and overall reactions, which were then documented by the interviewer and observers seated behind 1-way mirrors.

**Figure .**
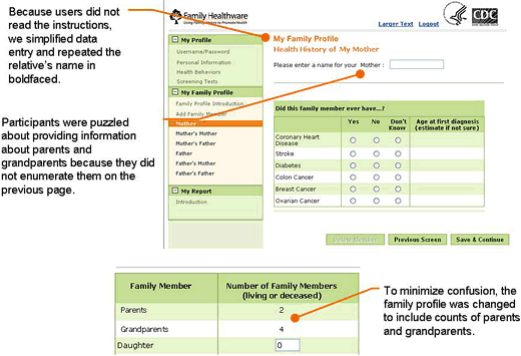
Example of a screen shot from Family Healthware usability testing, with notations of findings from usability testing.

The first round of usability testing focused primarily on the tool's navigation and data entry of family history and lifestyle and screening behaviors. Participants were generally positive about the concept of the tool and, for the most part, found it easy to use and navigate. Testing identified a number of design features that could benefit from minor changes to minimize errors and misunderstanding. For instance, we repositioned several key data entry points to be more prominent on the page. We moved error messages closer to the actual error and clarified the wording to facilitate correct data entry. We greatly simplified text, as we learned that users quickly navigated through the tool and tended not to read more than a few instructions or bulleted points. Users skimmed over or ignored paragraphs, and text even as short as a few sentences was not always read. Despite our best efforts to use standardized and validated questions for screening and lifestyle, we still encountered text we could improve. For example, our smoking question asked about cigarettes but did not include cigars. The revised question now asks, "Do you smoke tobacco, including cigarettes, a pipe, or cigars?"

After making all of the text changes, we retested the enhanced tool by focusing on the results that it generated, namely, the familial risk assessment for each disease and the lifestyle and screening message content. Users immediately focused on diseases for which they had a moderate or strong familial risk. Although we wanted (and even expected) users to carefully study their risk assessment for each disease, we quickly learned that they were most interested in the diseases where increased familial risk was identified. We also wanted feedback on how much information about health promotion and disease prevention to provide to users; thus, we tested 2 types of messages, a long text version of screening recommendations and a short bulleted-list version for lifestyle changes. Almost all users clearly preferred the short bulleted content. However, many participants wanted the messages and tool to be more comprehensive. For instance, instead of reading "lose weight" they wanted to know how much weight to lose. (We later included tables of body mass index [BMI] into the software to provide users with the ideal weight range for their height.) They also wanted the tool to be integrated with aspects of the medical record and history and to document the user's blood pressure measurements and cholesterol levels. Although we could not turn the tool into a comprehensive electronic medical record, we could respond to several suggestions. To balance information needs and preferences of our users, we developed a tiered approach to the messages. The first message that appeared on the screen contained a sentence or 2, with a clickable symbol to reveal a more detailed message. In addition, we heard we should praise healthy lifestyle choices, not just focus on what people were "doing wrong." Consequently, we added messages reinforcing positive behaviors.

## Results

Family Healthware is intended as a screening tool for population-wide use; therefore, the amount of data that is collected is limited to relevant and actionable information. Family Healthware does not collect the detailed and extensive family histories that genetics professionals need to evaluate people at risk for genetic disorders. As a screening tool, however, Family Healthware can identify people with a strong family history who might require further in-depth risk assessment by a specialist or another computerized model, such as BRCAPRO, a genetic susceptibility model that predicts the likelihood of a *BRCA1* or *BRCA2* gene mutation (48).

The data elements collected in Family Healthware include the following:

- Personal information: name, date of birth, sex, adoption status, race/ethnicity, Ashkenazi Jewish heritage, and current height and weight, which are used to calculate BMI.
- Health behaviors: smoking, physical activity, fruit and vegetable consumption, alcohol use, and aspirin use.
- Screening tests: blood cholesterol, blood pressure, blood glucose, fecal occult blood test, sigmoidoscopy, colonoscopy, and, for women, clinical breast exam and mammogram.
- Disease history of a person's first- and second-degree biological relatives (parents, siblings, children, grandparents, aunts, and uncles) for the 6 diseases included in the tool, with age at first diagnosis described by 5-year increments.

**Box. Screening message for an adult with moderate or strong familial risk of coronary heart disease or stroke who has not had a blood cholesterol test in the past 5 years, from Family Healthware computer-based health risk assessment software tool.**

Get your cholesterol tested. Talk to your health professional about your family history, how it affects your risk of coronary heart disease or stroke, and your options for screening and prevention.

Your cholesterol testing should include a measure of your total cholesterol, low-density lipoprotein (the "bad" cholesterol), high-density lipoprotein (the "good" cholesterol), and triglycerides. If your cholesterol levels are high or abnormal, changing your lifestyle and/or taking medication can reduce your risk of coronary heart disease and stroke. Because of your increased risk, you may need to test for other cardiovascular risk factors. Ask your health professional how often you should test your cholesterol. The frequency of testing will depend on your cholesterol levels, other risk factors, and if you already are being treated for cholesterol problems.

The report generated by the tool includes a statement about familial risk and disease-specific lifestyle and screening messages that are based on familial risk as well as a person's individual health and screening behaviors. For example, a person with a strong familial risk of diabetes who had never had a blood glucose test would receive the message, "You may benefit from blood sugar testing because of your family history. Talk to your health professional about your blood sugar and how it affects your risk of diabetes." Likewise, people who indicate that they are not exercising at the level currently recommended (49) would receive the message, "Increase your physical activity." Each of these short messages is linked to a longer explanation of why the behavior or screening test is important and what issues the users should discuss with their health care professional (Box). The challenge in delivering individualized behavioral and screening messages was collecting enough information to determine compliance with behavioral and screening recommendations by age, sex, and familial risk level — and not burdening the respondent with too many questions. The behaviors and screening tests included in Family Healthware are those that are associated with the 6 diseases in the tool and for which an evidence-based guideline exists (50,51).

### Validation of Family Healthware

The rules underlying the familial risk assessment and pedigree analysis functions of the FRS methods used in Family Healthware are derived from empirical data that have accumulated in the scientific literature over several decades (44). Validation of the risk stratification rules included in Family Healthware has been limited because few existing population-based data include all of the family history-related data elements and follow-up outcome data on the diseases of interest. Determining the clinical validity of the FRS rules and adjusting them accordingly is a critical component of a research agenda to evaluate Family Healthware and the use of family history in preventive medicine.

Recently, we assessed the performance of risk stratification rules similar to those used in Family Healthware for identifying the outcomes of early-onset (diagnosed before age 60) CHD and type 2 diabetes by using cross-sectional survey data from nationally representative samples (11,20,52). With data from the HealthStyles 2003 survey, we found that, after adjusting for demographics, compared with weak familial risk, strong and moderate familial risk categories were significantly associated with a 4.9-fold (95% confidence interval [CI], 3.3-7.2) and 2.0-fold (95% CI, 1.1-3.6) increase in early-onset CHD, respectively (11). Using the HealthStyles 2004 survey, we found that, compared with respondents with a weak familial risk for diabetes, respondents with strong and moderate familial risk had increased self-reports of diabetes (7.6-fold [95% CI, 5.9-9.8] and 3.6-fold [95% CI, 2.8-4.7], respectively) after adjusting for demographic factors (52). In a more recent analysis of 16,388 adults interviewed for the National Health and Nutrition Examination Survey during 1999-2004, we found that the odds of having diabetes for people in the strong and moderate familial risk categories, compared with those on the weak category, were 5.5 (95% CI, 4.4-6.8) and 2.3 (95% CI, 1.8-2.9) times higher, respectively (20). These studies support the use of a 3-tiered familial risk assessment method to identify people at increased risk for selected chronic diseases, but true validation of the FRS used in Family Healthware will require the assessment of sensitivity, specificity, and predictive value in large prospective cohorts.

Evaluating the usefulness of Family Healthware will require multiple studies in different populations and settings. Even if the risk assessment is valid and useful for predicting who will get disease, will the feedback and prevention messages improve health behavior and screening? As a first step in answering this question, 3 academic research centers — The University of Michigan School of Medicine, Evanston Northwestern Healthcare Research Institute, and Case Western Reserve University School of Medicine — are collaborating on a study set in primary care practices to examine the effect of Family Healthware on risk perceptions, disease-related attitudes and beliefs, and change in health behaviors. Results from this study are forthcoming and may shed light on the use of Family Healthware in a real-world setting and the effect of familial risk notification on attitudes about disease and motivation to change behaviors.

One goal of the initiative was to identify effective family history tools and strategies that could be used to identify people at increased risk for chronic diseases and influence their health behaviors and use of preventive services. Even though more work needs to be done to validate the risk algorithms, early studies (11,20,52) demonstrate the clinical validity of a 3-tiered familial risk gradient from weak to moderate to strong. The area in greatest need of study is whether familial risk assessment might influence health behaviors and use of preventive services. We hope that further research and evaluation will provide support for the use of new and innovative tools as part of an overall strategy to reduce common chronic diseases in the population.

## Figures and Tables

**Table.** Selected Studies Describing the Risk Association, Prevalence, and Accuracy of Self-Reports for Family History and the 6 Diseases Evaluated in Family Healthware

**Disease**	Disease Risk Associated With Family History	Prevalence of Family History of Disease	Accuracy of Reported Family History
Coronary heart disease (CHD)	Risk of cardiovascular disease (CVD) associated with sibling CVD (OR = 2.0) and parental CVD (OR = 1.5)^10^ 14% of Utah families with a family history of CHD accounted for 72% of early-onset (at age <55 y) CHD cases^2^	Approximately 50% of respondents to a national survey had a first- or second-degree relative with CHD^11^ 13% of women aged 45 or older had parental history of myocardial infarction^12^	Comparing study participant's report with relatives' report yielded 79% sensitivity and 91% specificity^13^ Comparing study participant's report with parents' report yielded 85% sensitivity and 93% specificity^14^
Stroke	Risk of large-vessel stroke associated with family history of stroke at age ≤65 y (OR = 2.2); also, risk of small-vessel disease (OR = 1.9)^15^ Risk of hemorrhagic stroke in women aged 18-44 y associated with family history of stroke in a first-degree relative (OR = 2.4); also, risk of ischemic stroke (OR = 1.8)^16^	26% of study participants (aged >45 y) reported a parental history of stroke, and 12% reported sibling history of stroke^17^	Comparing study participant's report with father's medical records yielded 42% sensitivity and 96% specificity; comparing study participant's report with mother's medical records yielded 51% sensitivity and 98% specificity^18^
Diabetes	Risk of diabetes associated with maternal diabetes (OR = 3.4), paternal diabetes (OR = 3.5), and both parents with diabetes (OR = 6.1)^19^ Risk of diabetes associated with strong (OR = 5.5) and moderate (OR = 2.3) familial risk as compared with weak familial risk^20^	31% of adults reported a family history of diabetes in a first-degree relative^21^ 48% of adults reported a family history of diabetes among first-degree relatives or grandparents^22^	Comparing study participant's report with parents' report yielded 87% sensitivity and 98% specificity^14^ Comparing study participant's report with sibling's report yielded 72% sensitivity and 98% specificity^14^
Breast cancer	Pooled estimate of RR associated with various family histories: any relative (RR = 1.9); a first-degree relative (RR = 2.1); mother (RR = 2.0); sister (RR = 2.3); mother and sister (RR = 3.6); and a second-degree relative (RR = 1.5)^23^ Risk for breast cancer associated with increasing numbers of affected first-degree relatives: 1 (RR = 1.8); 2 (RR = 2.9); ≥3 (RR = 3.9)^24^	5%-10% of women with breast cancer have a mother or sister with breast cancer and up to 20% have a first- or second-degree relative with breast cancer^23^ 13% of women with breast cancer and 7.3% of controls reported ≥1 first-degree relatives with a history of breast cancer^24^	Comparing patient's report with first-degree relatives' medical record or self-report yielded 95% sensitivity and 97% specificity^9^ Comparing participant's report with registry data for first-degree relatives yielded 82% sensitivity for controls and 85% sensitivity for cases^25^
Ovarian cancer	Lifetime probability of ovarian cancer increases from about 1.6% for a 35-year-old woman without a family history of ovarian cancer to about 5% if she has 1 relative with ovarian cancer and 7% if she has 2 affected relatives^26^ Risk of ovarian cancer associated with breast cancer in first- or second-degree relative (RR = 1.4); also, ≥2 first-degree relatives with breast cancer (RR = 1.8)^27^	1.8% of adult respondents to a national survey reported having ≥1 first-degree relative with ovarian cancer^28^	Comparing patient's report with first-degree relatives' medical record or self-report yielded 83% sensitivity and 99% specificity^9^ Comparing participant's report with registry data for first-degree relatives yielded 50% sensitivity for controls and 67% sensitivity for cases^25^
Colorectal cancer (CRC)	Risk of CRC associated with affected first-degree relatives (RR= 1.7); also, with ≥2 affected first-degree relatives (RR = 2.8)^29^ Pooled estimates of relative risk for CRC by affected relative: a first-degree relative (RR = 2.25); parent (RR = 2.26); sibling (RR = 2.57); ≥1 relative (RR = 4.25)^30^	5% of adult respondents to a national survey reported having ≥1 first-degree relative with CRC^28^ In a large cohort study of health professionals, 9.4% of men reported a history of CRC in a parent or sibling and 10% of women reported the same^29^	Comparing patient's report with first-degree relatives' medical record or self-report yielded 90% sensitivity and 97% specificity^9^ Comparing participant's report with registry data for first-degree relatives yielded 81% sensitivity for controls and 65% sensitivity for cases^25^

Abbreviations: OR, odds ratio; RR, relative risk.
